# Antiproliferative, Apoptotic Effects and Suppression of Oxidative Stress of Quercetin against Induced Toxicity in Lung Cancer Cells of Rats: *In vitro* and *In vivo* Study

**DOI:** 10.7150/jca.52088

**Published:** 2021-06-26

**Authors:** Zhaorigetu, Islam M Farrag, Amany Belal, Manal H. Al Badawi, Alsayed A. Abdelhady, Fawzy M. A. Abou Galala, Abdou El-Sharkawy, Asmaa A. EL-Dahshan, Ahmed B. M. Mehany

**Affiliations:** 1 Thoracic Surgery, Inner Mongolia People's Hospital, Hohhot City, Inner Mongolia Autonomous Region, 010020, China.; 2 Forensic Medicine & Clinical Toxicology Department, Faculty of Medicine for Girls, Al-Azhar University, Cairo, Egypt.; 3 Department of Pharmaceutical Chemistry, College of Pharmacy, Taif University, P.O. Box 11099,Taif 21944, Saudi Arabia.; 4 Department of anatomy, Faculty of Medicine, Helwan University, Helwan, Egypt.; 5 Department of anatomy, Faculty of Medicine, Al-Azhar University, Cairo, Egypt.; 6 Department of zoology Faculty of science (Girls branch), Al-Azhar University, Cairo, Egypt.; 7 Department of zoology Faculty of science, Al-Azhar University, Cairo, Egypt.

**Keywords:** lung cancer, quercetin, cyclophosphamide, cell cycle, molecular docking, antioxidant enzymes

## Abstract

In the present study, quercetin was examined against lung human cancer cells using A549 and H69 cancer cell lines in addition to normal non cancer cells (W138). Two genes Bax and Bcl-2 that play an important role in apoptosis pathways were investigated. Also Immunohistochemical study for caspase-3 which is considered as indicator for apoptosis was performed. Quercetin showed good anti proliferative activity against tested lung cancer cell lines, IC_50_ values on A549 are 8.65, 7.96 and 5.14 µg/ml at 24, 48 and 72h respectively. Also significant effects of quercetin on Bax, Bcl-2 and caspase-3 were observed, that can prove its ability to induce apoptosis. On the other hand quercetin showed good therapeutic effects against cyclophosphamide induced lung toxicity that were observed in the histopathology study. *In vitro* studies were also performed such as cell cycle analysis through flowcytometry. The obtained results from all these performed analysis proved that quercetin can induce apoptosis in human lung cancer cells, additionally quercetin showed ability to reduce MDA and increase SOD and GSHP levels which indicates its ability in suppressing oxidative stress, Quercetin has played a therapeutic role in cyclophosphamide induced lung toxicity as it has improved restoring of the damaged lung tissue as discussed in this research work.

## Introduction

Lung cancer is a major cause of tumor-related deaths in humans worldwide; otherwise there is ability to prevent tumors by controlling weight, increase vegetarian eating, Physical activity, reducing alcohol and tobacco according to WHO reports 1. Humans that have a 5-year survival rate are less than 15% 2. As reported Non-small cell lung cancer (NSCLC) has largely formed heterogeneous group of tumors and accounts for about 85% of all lung cancers 3. Recent studies have reported that surgical removal of the lung is not effective in tumor's treatment. Also, chemotherapy alone or in combination with radiation is good in treatment strategy for lung cancer 4, 5.

There are numbers of antitumor drugs which were approved for cancer treatment; they were derived from natural products 6. Natural products have to be considered as anticancer active agents because their toxicity is very low. One of these natural products is quercetin (QEU), that have showed multiple pharmacological properties for example anti-inflammatory, antioxidant, antiatherosclerosis, anti-apoptotic and antiproliferation activities 7. QEU has a good activity in some diseases with high potential effect against cancer, a lot of vegetables and fruits which human take in food daily contain a good quantity of QEU 8. *In vitro* and *in vivo* studies illustrated that QEU has antitumor activity 9 and was reported to prevent cancer induced by chemotherapeutic agents in experimental studies 10. QEU is reported to inhibit tumor growth in colon cancer 11. Other studies suggest that quercetin inhibits the reactive oxygen species inside the cell and can cause programmed cell death for the cancer cells 12. Former studies also postulated that QUE ameliorated cyclophosphamide-induced cardiotoxicity, urotoxicity, and genotoxicity in mice 13.

Cyclophosphamide (CYP) is a notable alkylating agent widely used alone or with other drugs in the treatment of chronic and acute leukemias, multiple myeloma, lymphomas. It is additionally used as an immunosuppressant for the treatment of rheumatoid arthritis, nephrotic syndrome, systemic lupus erythromatosis and bone marrow transplantation. In spite of its vital role in chemotherapy, cyclophosphamide produces serious effects including; hepatotoxicity, nephrotoxicity and lung toxicity 14. As per the International Agency for Research on Cancer (IARC), CYP is widely used as reference mutagen and is classified as carcinogenic for both animals and humans 15.

Phosphoramide mustard and acrolein are the two active metabolites of CYP 16. Phosphoramide mustard is responsible for the antineoplastic effects of CYP, while its toxic effects are linked to acrolein 17. Acrolein, is responsible for chemically alkylating DNA as well as proteins, resulting in cross-links and thereby resulting in cytotoxicity 18.

Experimental evidence proposes that the occurrence of oxidative stress after CYP administration prompts decrease in the activities of antioxidant enzymes and increase in lipid peroxidation (LPO) level in liver and lung of mice 19, 20. Alongside these, because of the absence of aldehyde oxidase and aldehyde dehydrogenase which are two key detoxifying enzymes, that catalyze the conversion of toxic aldehydes to less toxic carboxylic acids, in the lung tissues, CYP has been accounted to cause selective pulmonary injury 21. CYP has a synergistic effect of matrix degrading proteases and adhesion proteins that enhances the process of pulmonary metastasis 22.

Free radicals cause peroxidation of lipid in cell components, leading to disruption of normal functions, results in stimulation of signaling pathways and subsequently increased apoptosis 23. Alveolar epithelial type II cells are particularly more susceptible to the deleterious effects of oxidants 24. Hence, lung damage is thought to be mediated by the generated reactive oxygen or nitrogen species and lipid peroxide formation in lung tissues. Moreover, elevated ROS production in this manner causes activation of cells of pulmonary defense system like neutrophils, monocytes, and macrophages resulting in pulmonary fibrosis 25.

Epidemiological studies have also reported CYP-induced pulmonary injury in humans. A comprehensive retrospective study has specified six patients over 20 years. In those patients, CYP was identified to be the sole factor that contributed to pulmonary injury. Clinically CYP-induced pulmonary toxicity was manifested by dyspnea, fever, cough, parenchymal infiltrates, gas exchange abnormalities, and pleural thickening 26. Taking into account the drawbacks of chemotherapeutic agents, there is a critical need to develop new treatment strategies that can limit or antagonize the toxicity of CYP.

Reported results suggest that antioxidant supplementation can reduce the side effects of chemotherapeutic agents 27. On the other hand cyclophosphamide causes oxidative stress and produces reactive oxygen species (ROS) 28. Cyclophosphamide induces many pathological effects of lung injury; as it promotes lung toxicity that may lead to damage of lung cells 29. It was reported that cyclophosphamide induced hepatotoxicity and caused DNA damage of hepatocytes 18, 30. Cyclophosphamide also causes toxic effects on liver and lung because it inhibits lung and liver cells to defense against free radicals 31, 32. Cyclophosphamide caused lung toxicity due to its ability to decrease detoxification enzymes 33. The present study aimed to detect the antiproliferative and apoptotic effects of quercetin against lung cancer cells and to detect the *in vivo* therapeutic effect of quercetin in cyclophosphamide induced lung toxicity. Moreover, molecular docking studies will be performed to investigate quercetin binding into oxidative stress enzymes.

## Materials and methods

### *In vitro* studies

#### Cell culture

The human lung cancer cell lines were obtained from ATCC, then cultured in RPMI-1640 medium (Gibco; Thermo Fisher Scientific, Inc., Waltham, MA); containing 1% penicillin/streptomycin (Hyclone; GE Healthcare Life Sciences, Logan, UT) and 10% fetal bovine serum (FBS; Gibco) in moist air with 5% CO2 at 37 °C. The study was performed as cells reached to 80% confluence.

#### Anti-proliferation assay

Anti-proliferation was examined using MTT assay, cells were seeded into 96-well plates at 2 × 10^4^ cells per well and incubated for 24 h. Cells were treated by 100, 50, 25, 12.5, 6.25 µg from quercetin then after treatment the cells were incubated for 24, 48, 72 h. then 0.05 mL of MTT solution (final concentration of 1 mg/mL) was added to each well and incubated for 4 h. The MTT solution was then removed, and 0.05 mL of acidified isopropanol (with 0.4% HCl) was added to solubilize the formazan crystals. Three replicates were performed, and at 570 nm they were analysed using a microplate reader (BD Biosciences, Mountain View, CA, USA). The ratio of viability (%) was calculated as OD sample/OD control × 100%.

#### Gene expression detection (BAX and Bcl-2)

A549 cells were grown in RPMI 1640 containing 10% fetal bovine serum at 37 °C, to be tested for BAX and Bcl-2. Cells were treated with the IC_50_ value of the tested drug, and then cells were lysed with cell extraction buffer. It was diluted in standard buffer over the range of the assay and measured for activity of BAX and Bcl-2 contents (cells are plated in a density of 2 × 10^3^ cells/well in a volume of 100 μl complete growth medium + 100 uL of the extracts per well in a 96-well plate for 48 h before enzyme assay using Invitrogen ELISA Kit (Cat. No. KHO1091) and DRG® Human Bax ELISA (EIA-4487) kit. Assays were repeated twice.

#### Cell cycle analysis

A549 cells at a density of 6 × 10^6^ cells in T 75 company flasks were treated with IC_50_ value in µg/ml for 48h The cells were harvested by trypsinization, fxed with 70% (v/v) ethanol overnight at -20 °C and washed with PBS and then labelled with propidium iodide (50 μg/mL) and incubated at RT in the dark for 30 min. Samples were analysed with a FAC Scan flow cytometer (BD Biosciences, Mountain View, CA, USA), and the percentages of cells in the G1, S, and G2/M cell cycle phases were investigated using ModFIT software.

#### Annexin V/PI analysis

A549 cells were stained using an Annexin V-FITC detection kit. Cells in the exponential growth phase were seeded in 60-mm^2^ dishes, incubated at 37 °C for 24 h, pretreated with Z-VAD-FMK (20 μM) and SP600125 (10 μM) for 2 h, and then treated with the obtained IC_50_ values at 48h. The cells were then collected, washed twice with PBS, resuspended in 400 μL of Annexin binding buffer, stained with 5 μL of Annexin V-FITC and 10 μL of PI, and incubated for 15 min at RT in the dark. The stained cells were analyzed with a FAC Scan flow cytometer (BD Biosciences, Mountain View, CA, USA), and the percentages of apoptotic cells were investigated using Flow Jo software.

### *In vivo* study

Male Swiss albino rats were obtained from animal house of faculty of science (220-250 g body weight), they were housed under 12:12 h light: dark cycles, at standard temperature (20 to 25 °C) and humidity (60 ± 10%) conditions, with food and water. A total number of 30 animals were randomly divided into three groups in the animal house. Group 1 was a control group, Group 2 received a single-dose intraperitoneal (i.p.) cyclophosphamide (200 mg/kg) on the 7^th^ day of the study. Group 3 was given, single-dose intraperitoneal (ip) CYP (200 mg/kg) (Cyclophosphamide was purchased from Sigma company), then after one week quercetin was administered i.p. with doses of 100 mg/kg for 15 days. On the 16 day of the experiment, the rats in all groups were anesthetized, all animals were sacrificed. The lung tissue samples were collected for histopathological examination.

#### Histopathological assay

The formalin preserved experimental Albino rat specimens; lungs were processed in an automated tissue processor. The processing consisted of an initial 2 steps; fixation and dehydration. Fixation comprising tissue immersion in 10% buffered formalin for 48 hours, followed by removal of fixative in distilled water for 30 minutes. Dehydration was then carried out by running the tissues through a graded series of alcohol, initially exposed to 70% alcohol for 120 minutes followed by 90% alcohol for 90 minutes and then two cycles of absolute alcohol, each for one hour. Dehydration was then followed by clearing the samples in several changes of xylene. It consisted of tissue immersion for an hour in a mixture comprising 50% alcohol and 50% xylene**,** followed by pure xylene for one and a half hours. Samples were then impregnated with molten paraffin wax, then embedded and blocked out. Paraffin sections (4-5 μM) were stained with hematoxylin and eosin 34. Stained sections were examined for inflammatory reactions, circulatory disturbances, degenerative, necrotic, apoptotic changes and any other pathological lesions in the examined tissues.

### Immunohistochemical studies

The demonstration of antigen in tissues by immunostaining is a two-step process. The first step is binding of the primary antibody to the related antigen, followed by visualization of this reaction or by linking a secondary antibody to which different enzyme systems are attached, collectively known as the universal. The primary antibody determines the specificity of the reaction; whereas, the secondary antibody, with its linked enzyme, causes amplification of the reaction, hence, increase of the sensitivity of the test. The Biotin-Streptavidin (BSA) system was used to visualize the markers. Diaminobenzidine (DAB) was used as chromogen since it allows a permanent preparation. Hematoxylin counter stain was used.

### Molecular docking studies

Pdb files were retrieved from protein data bank, refined, protonated and water molecules were removed, site of binding was detected by site finder in MOE software. Quercetin molecule was drawn by MOE builder and saved as mdb file. Swissdock was used for predicting the binding of quercetin with SOD (supplementary data) and MOE was used to predict binding of quercetin with glutathione peroxidase enzyme.

### Oxidative parameters determination

Oxidative stress parameters (superoxide dismutase (SOD), malondialdehyde (MDA) and glutathione peroxidase (GSHP). were evaluated in tissues of lung using an ultraviolet spectrophotometer (UV-2410PC model, Shimadzu, Japan) using commercial diagnostic kits (Nanjing Jiancheng Biotechnology Co., Ltd, Jiangsu, China).

## Results

### Anti-proliferation assay

MTT assay was used to examine the effect of quercetin on A549 and H69 cancer cells, the results showed a great reduction of cell viability as shown in Table 1, after 24h, 48h and 72 hours, IC_50_ values of quercetin against A549 are 8.65, 7.96 and 5.14 µg/ml respectively while IC_50_ values of quercetin against H69 are 14.2, 10.57 and 9.18 respectively.

### Gene expression (BAX and Bcl-2)

Table 2 is representing the effects of quercetin on Bax and Bcl-2 concentrations when compared to the control. Quercetin has increased the expression of Bax more than 7 times of its expression in the control cells (51.48). Also there is a high decrease in Bcl-2 concentration from 5.058 for the control to become 1.947 after treatment with quercetin.

### Cell cycle analysis

**Figure 1:**
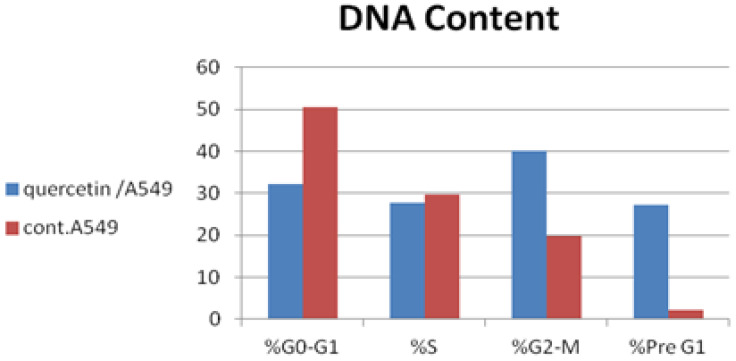
DNA content in A549 cells after treatment with quercetin.

Data presented in the Table 3 and Figure 1 demonstrated that quercetin caused cell cycle arrest at G2/M phase, the percentage of A549 cells at that phase was 40.15% after treatment with quercetin, however the percentage was 19.87% in the untreated control cells. Also it is noticed that the percentage at pre G1 phase was increased after treating cells with quercetin to become 27.31%, when compared with 2.19% for the untreated cells, this indicates its high ability in promoting apoptosis. Figure 1 also illustrated the DNA content in the cells at different phases.

### Annexin V/PI analysis

**Figure 2:**
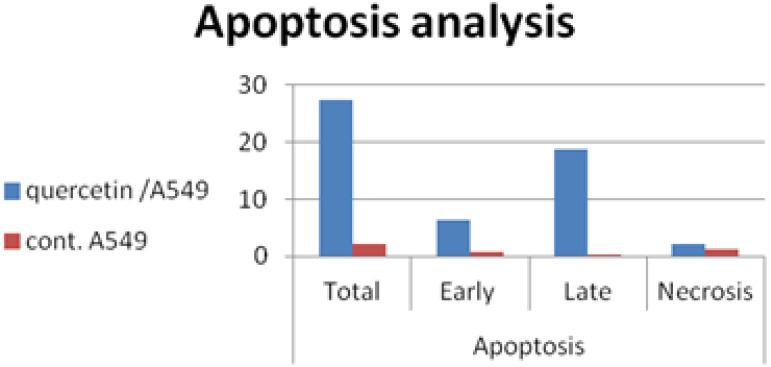
Early and late apoptosis in A549 cells after treatment with quercetin.

To examine if cell death that occurred in pre G1 phase is related to apoptosis or necrosis we have performed annexin V/PI analysis. The obtained results are represented in Table 4 and Fig. 2 quercetin induced apoptosis in both early and late stage.

### *In vivo* study

#### Histopathological study

##### Group 1: Control

**Figure 3:**
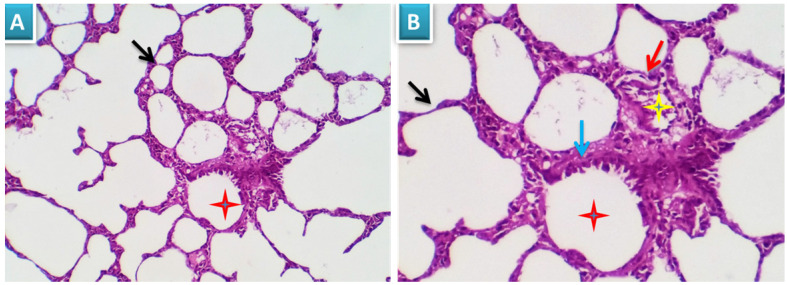
Photomicrograph from lung of group 1 showing normal histomorphological structures with preserved features of bronchial mucosal and submucoasal cellular features (red star and blue arrow). The peribronchial and interalveolar blood vessels are apparently normal (yellow star). The alveolar epithelial lining comprising the pneumocytes type I and II, the alveolar macrophages (pneumocyte type III) and perialveolar intersitium are of normal population and structure (black arrows). H&E X 100 (A), 200 (B).

Examined sections revealed apparently normal bronchial and bronchiolar histomorphological structures with maintained features of mucosal and submucoasal cellular features. Mininmal peri-bronchiolar lympho-plasmacytic infiltration were seen. The peribronchial and interalveolar blood vessels were apparently normal. The alveolar epithelial lining comprising the pneumocytes type I and II, the alveolar macrophages (pneumocyte type III) and perialveolar intersitium were of normal population and structure (Fig. 3A & B).

##### Group 2: Cyclophosphamide group

**Figure 4:**
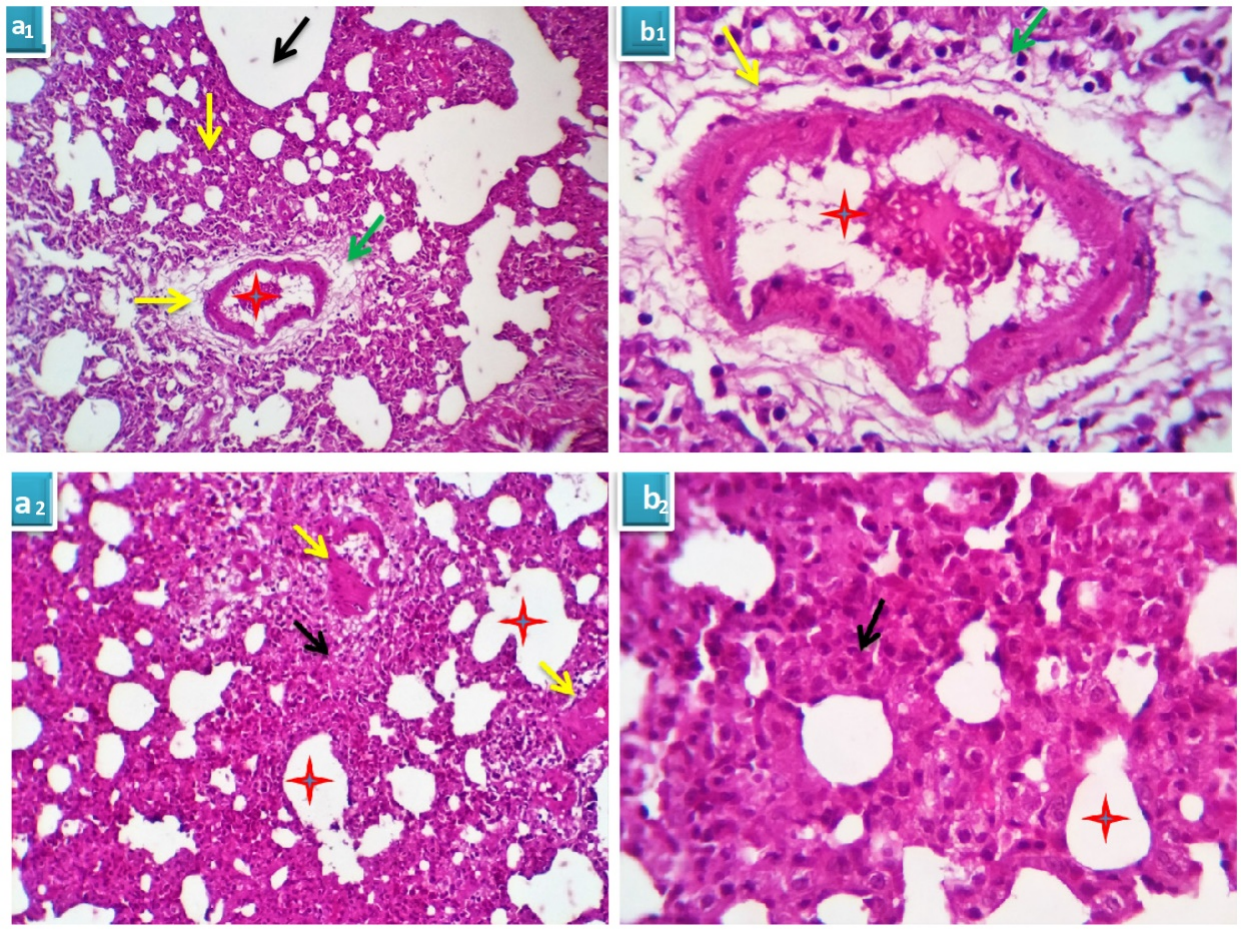
** A.** Photomicrograph from lung of group 2 showing perivascular edema (yellow arrows) with round cells infiltration (green arrows), destruction of the vascular intima with thickening and hyalinization of the vascular walls (red stars). H&E X 100 (a_1_), 400 (b_1_). **B.** Photomicrograph from lung of group 2 showing focal pneumonic areas with replacement of the alveoli by large number of round cells and macrophages (black arrows). Compensatory focal alveolar emphysema is seen (red stars). H&E X 100 (a_2_), 400 (b_2_).

Serial sections from lung of this group pointed out moderate perivascular edema with round cells infiltration, destruction of the vascular intima with thickening and hyalinization of the vascular walls (Fig. 4A) beside multifocal pneumonic areas with replacement of the alveoli by large number of round cells and macrophages. Compensatory focal alveolar emphysema was also seen (Fig. 4B).

##### Group 3: Cyclophosphamide and Quercetin

**Figure 5:**
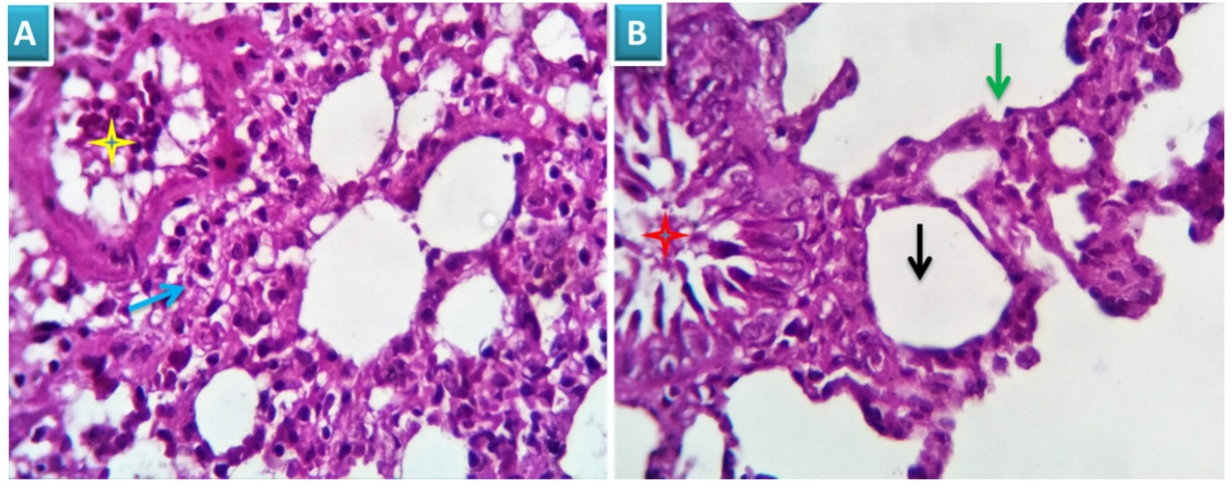
Photomicrograph from lung of group 3 showing healing and remodeling process with presence of remnant of inflammatory cells in the interstitial tissue (blue arrow). The bronchial and alveolar structures are normal (green arrow and red star). The blood vessels resume apparently healthy intimal and medial structures (yellow star). Focal alveolar emphysema was still seen (black arrow). H&E X 100 (A), 400 (B).

The pulmonary tissue underwent healing and remodeling process with presence of remnant of inflammatory cells in the interstitial tissue. The blood vessels washed out the toxic product and resumed apparently healthy intimal and medial structures. The bronchial and alveolar structures were apparently normal. Focal alveolar emphysema was still seen in some area of the pulmonary parenchyma (Fig. 5).

#### Immuno-histochemical results for caspase 3

##### Group1: control

**Figure 6:**
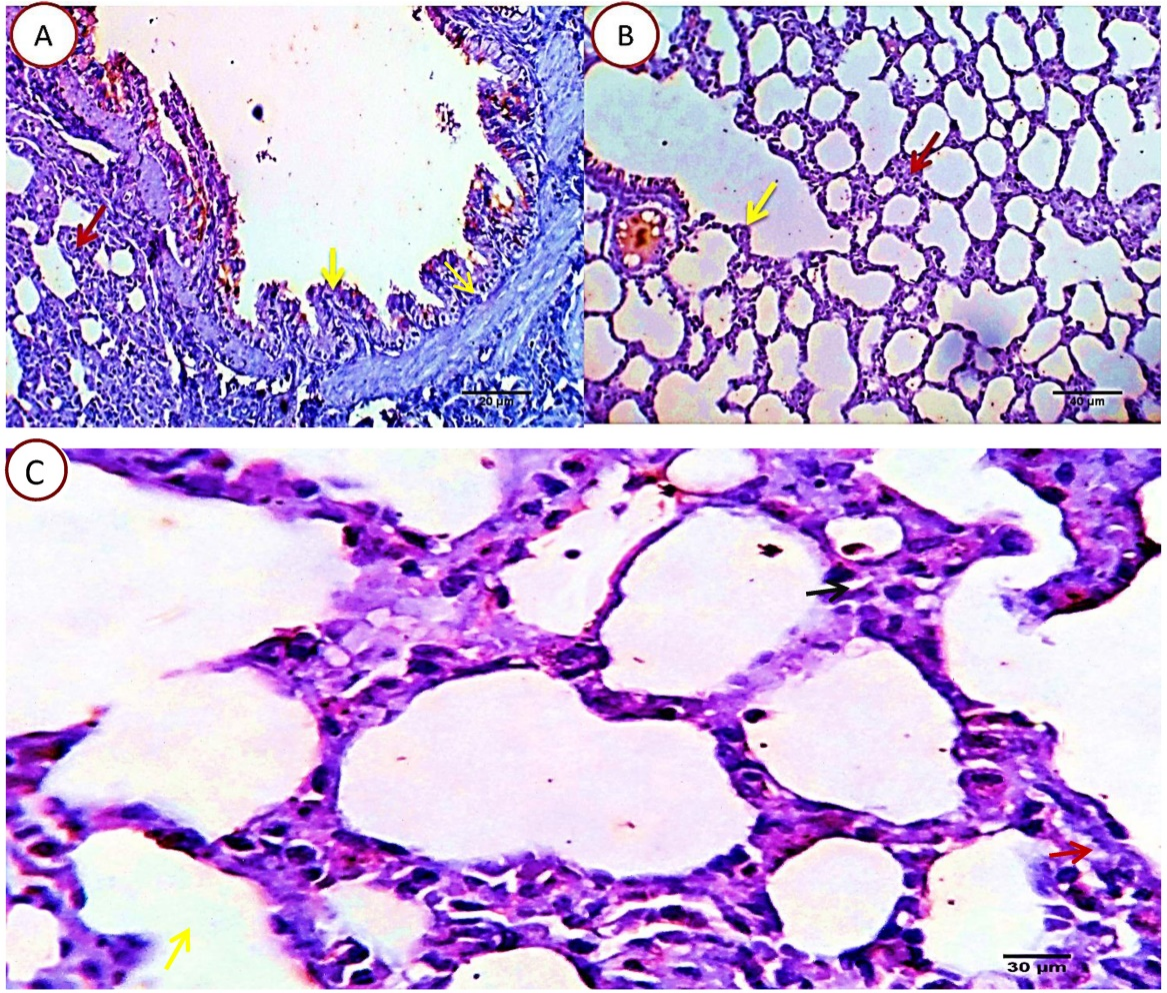
Photomicrograph from lung of normal (control rats), immune-stained with Caspase -3, showing very mild positive cytoplasmic dark brown staining reaction in the pneumocytes type 1 and 2 (2, yellow and red arrows) and in the alveolar macrophages (2, black arrow). Very few bronchogenic cells are uncreatively Caspase positive (1, yellow arrow). Scale bars 30, 40, 30 um.

Lung sections revealed about 4-6%/HPF (average 5.2%) of pneumocytes type 1 and 2 and alveolar macrophage cells positive for caspase 3 apoptotic marker signal, the remaining tissue cells were normal. Positive cells showed characteristic brownish cytoplasmic stainability. Bronchial and bronchiolar epithelial cells were negative for Caspase 3, however it seems that some bronchogenic cells with mucinous productive capacity (mucin producing cells) undergo programmed cell death following pouring of their mucin contents in the bronchial lumen with a resulting unrelated positive apoptotic reactions (Fig. 6).

##### Group 2: Cyclophosphamide group

**Figure 7:**
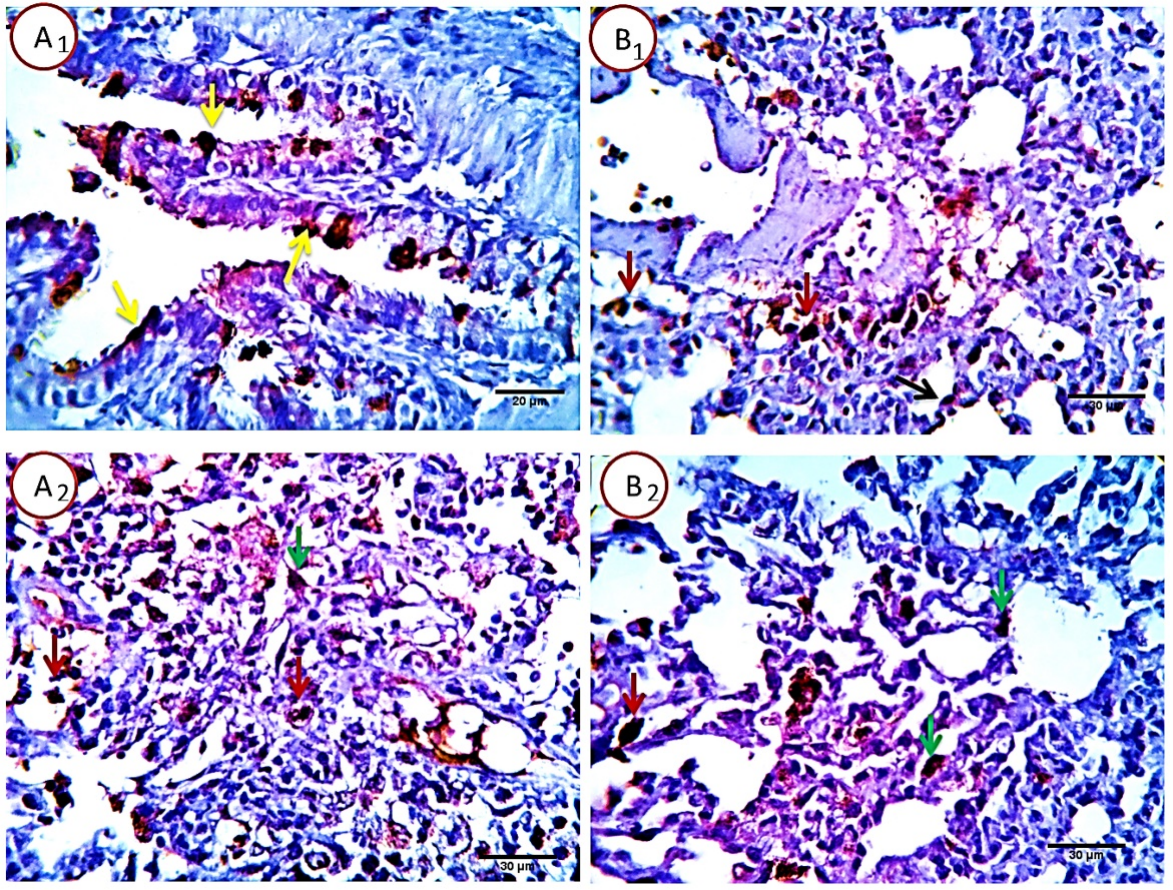
Photomicrograph from lung of Cyclophosphamide treated group, immune-stained with Caspase-3, showing mild to moderate proportional numbers (27-30 %) of cells positively reactive to caspase-3, with the more pronounced effect in the alveolar macrophages (red arrows) and pneunocytes type 2 (green arrows). Pneumocytes type 1 (black arrow) and some bronchogenic cells (relative and un-relative) are also positively stained. Scale bars 20, 30 um.

Serial sections from lungs of this group pointed out a dramatic apoptotic changes in all of the pulmonary histologic structures as the bronchial epithelium, alveolar cells of pneumocytes type 1 and 2 beside the alveolar macrophages, all were affected with a mild to moderate proportional numbers 27-30% HPF (average 28.8%) of the morphometric analyzed cells, they were positively reactive to caspase -3, with the more pronounced effect in the alveolar macrophages and pneunocytes type 2, postulating an additional immune-depressive effect of the cyclophosphamide beside its real cytotoxic effect (Fig. 7).

##### Group 3: Cyclophosphamide and Quercetin

**Figure 8:**
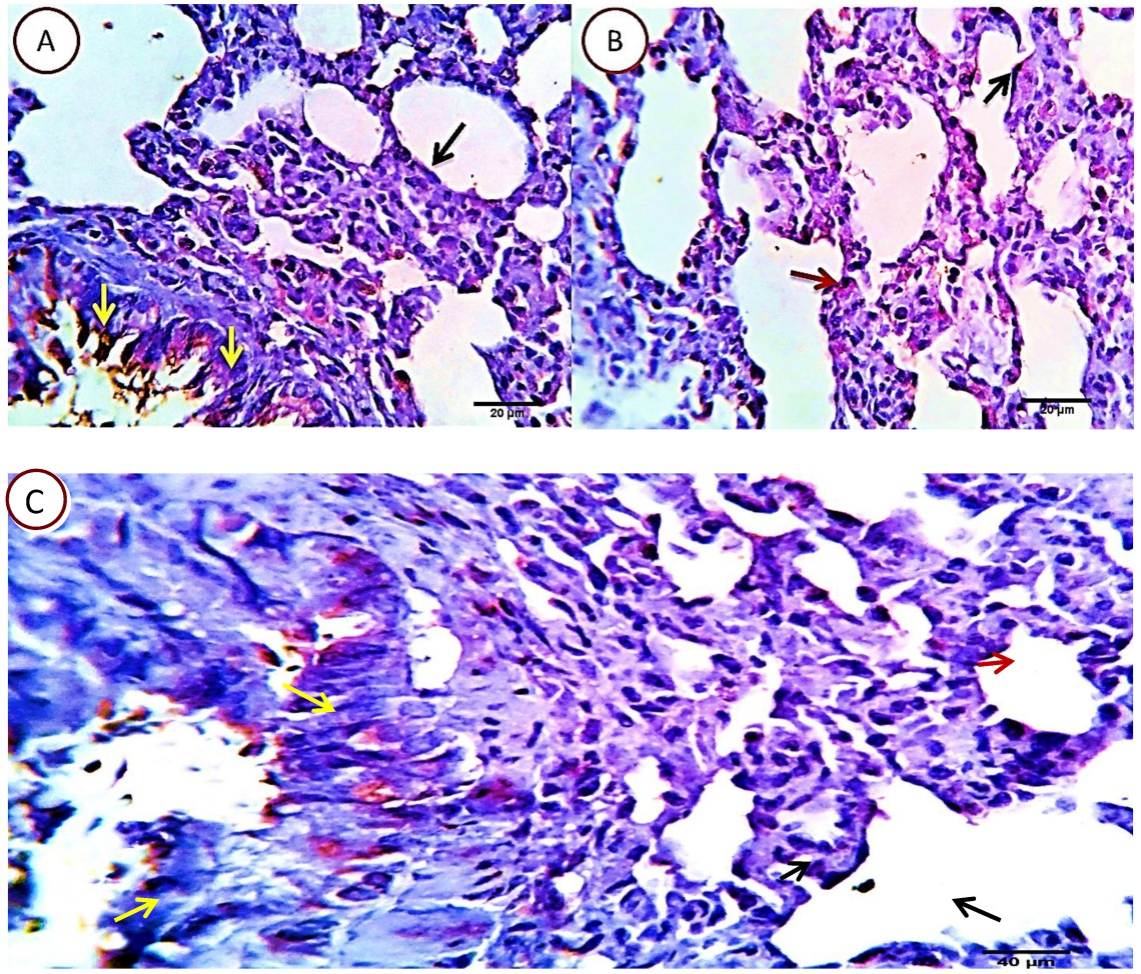
Photomicrograph from lung of (Cyclophosphamide and Quercetin) immune-stained with Caspase-3, showing very low (5-7%) of the cells are positively stained. The pneumocytes type 2 are the most affected cells, they are seen related to the alveolar wall (red arrow) and the interalveolar tissue. Other cells including bronchial (yellow arrows), bronchiolar and alveolar cells (pneumocytes type 1) (black arrow) appears normal or sparsely affected, especially the unrelated positively stained bronchial epithelial cells (yellow arrows). Scale bars 20, 40 um.

Serial sections from lungs of this group pointed out an optimizing protective effect of the used quercetin as the apoptotic index in most of the pulmonary tissue was very low. Nearly, 5-7% HPF (average 7%) of the morphometrically analysed cells were positively reacted, with the pneumocytes type 2 as the most affected cells, they were seen related to the alveolar wall and the interalveolar tissue. Other cells including bronchial, bronchiolar and alveolar cells (pneumocytes type 1) were normal or sparsely affected, especially the unrelated positively stained bronchial epithelial cells (Fig. 8).

#### Molecular docking studies of Quercetin with anti-oxidative stress enzymes (GSPH and SOD)

**Figure 9:**
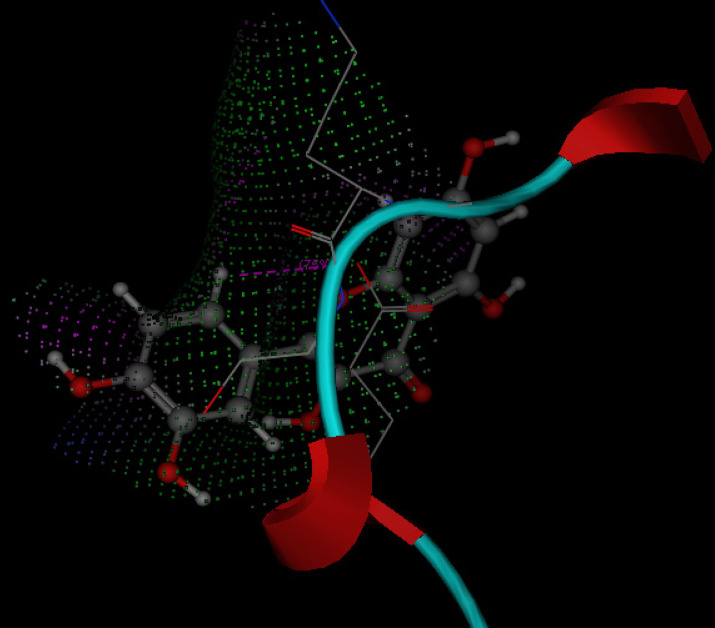
3D mode for quercetin inside GPx (Pdb ID: 5Q5H).

Molecular docking studies are performed in this research work to investigate if Quercetin has a good ability to bind with these enzymes, mode of binding, possible interactions and binding score. 1PM9 is the pdb file for SOD, it was retrieved from protein data bank (https://www.rcsb.org/structure/1PM9) as pdb file then uploaded to swissdock server (http://www.swissdock.ch/) with Quercetin to perform docking process, the obtained results revealed the ability of quercetin to bind to SOD with full fitness score equals -1876.34 kcal/mol and estimated ΔG = -8.2 kcal/mol which indicate promiscuity of quercetin to target and affect SOD enzyme (supplementary data). Additionally 5H5Q is the pdb file for glutathione peroxidase enzyme (https://www.rcsb.org/structure/5H5Q), it was also retrieved from protein data bank and used for docking with quercetin using MOE program, the obtained results revealed that quercetin has the ability to fit at the binding site of glutathione peroxidase enzyme with docking score energy equals -11.64 Kcal/mol (Fig. 9). These obtained in silico studies indicated the ability of quercetin to affect anti-oxidant enzymes and encouraged us to investigate the ant- oxidative stress activity of quercetin on SOD and GSPH to support molecular docking results.

### Oxidative parameters determination

To determine the antioxidant effect of Quercetin, MDA and antioxidant enzymes (SOD and GSHP), they were evaluated in the lung of the control, CP-intoxicated lung of rats and after treatment of the CP-intoxicated lung with quercetin. The results are represented in Table 5, they showed decrease in GSHP and SOD and increase in MDA in intoxicated lung than the control. However after treating the intoxicated lung with quercetin the obtained results revealed increase in both GSHP and SOD and showed dramatic decrease in lung MDA to be nearly equal to the control as shown in the following table. From the obtained results we can conclude that Quercetin suppresses oxidative stress in induced cyclophosphamide lung intoxication of rats.

## Discussion

The aim of the present study was to detect the activity of quercetin against lung cancer cells. IC_50_ values revealed that quercetin has a good anticancer efficacy. Recent studies have reported that quercetin possess anticancer activity 35. In the present study we focused on quercetin efficacy in treatment of human lung cancer cell lines and the results showed good inhibition of cell growth in MTT assay method. Therefore, we exposed A549 cells to further investigations to explore the mechanism of action of quercetin in cancer cell proliferation and the results that derived from these analysis showed that quercetin has induced apoptosis of cancer cells by increasing the level of expression of Bax gene and decrease the expression of Bcl-2. Caspases are known as a family of cysteine proteases that are required for cytokine maturation and apoptosis execution, they have been suggested to contribute to both apoptotic 36 and non-apoptotic cellular phenomena 37. The effector caspases are activated through proteolytic cleavage more than that expected by the initiator caspases. Present data showed that caspase 3 are activated by quercetin in the apoptotic pathway. The obtained data have proved quercetin's ability to induce apoptotic pathway. Activation of caspase-3, will result in cell death 38. In the present study, cell cycle analysis was performed to investigate the inhibition of the cancer cell cycle phases and apoptosis induction. In our study, cell cycle of A549 cells showed cell arrest at both S and G2-M phases. Wiśniewska et al. (2017) 38 have reported the effect of quercetin on the essential cytoskeletal elements, such as microfilaments, microtubules, cytoskeleton-driven processes and vimentin intermediate filaments in A549 non-small cell lung cancer cells. Their results pointed out that quercetin induced apoptosis via the regulation of BCL2/BAX, also necrosis and mitotic catastrophe, and suppressed the migration of those cells. The reduced migration of A549 cells detected with quercetin therapy might be due to the disassembling influence of quercetin on vimentin filaments, microtubules, and microfilaments associated with its suppression effect on N-cadherin and vimentin expression.

On the other hand the present study investigated the therapeutic effect of quercetin on cyclophosphamide induced lung damage and evaluated the parameters of immuno and histopathological examinations. Examination of lung tissues in the cyclophosphamide treated group suggested a dramatic apoptosis of bronchial epithelium, alveolar cells of pneumocytes type 1 and 2 beside the alveolar macrophages, all were affected with a mild to moderate proportional numbers 27-30% HPF (average 28.8%) of the morphometric analyzed cells were positively reactive to caspase-3 enzyme, with the more pronounced effect in the alveolar macrophages and pneumocytes type 2, postulating an additional immune-depressive effect of the cyclophosphamide beside its real cytotoxic effect. On the other hand, an optimizing protective effect was observed in quercetin treated rats as the apoptotic index in most of the pulmonary tissue was very low. The anti-inflammatory impact of QUE causes inhibition of inflammatory mediators and affects immunity and the inflammation response.

These findings were supported by a study that illustrated decreasing and changes in the epithelial cell components and alveolar capillary, lymphocytic interstitial pneumonia, extensive fibrous tissue proliferation, and thickened alveolar septa due to cyclophosphamide administration 39, while another study reported degenerated alveolar cells, morphological changes in the alveolar septa, cells of the alveolar lumen of cyclophosphamide group, whereas quercetin treatment diminished the degeneration and inflammatory changes in the CYP group 40. So the obtained data of the present study demonstrated that using of quercetin in treatment has helped in decreasing CYP induced lung toxicity and maintained normal lung functions and lung structure. Molecular docking studies proved the ability of quercetin to bind to oxidative stress enzymes SOD and GSPH this studies were supported by the obtained results from evaluating oxidative stress parameters as shown in Table 5.

## Conclusion

From the obtained results in this work, we can conclude that quercetin have good and remarkable anticancer activity against human lung cancer cells. Quercetin showed inhibitory effect on lung cancer cell proliferation, by inducing apoptosis through BAX, BCL-2 and caspase3. Quercetin caused G2-M phase arrest in lung cancer cells. Also quercetin treatment has shown therapeutic effect after cyclophosphamide induced lung toxicity in rats. The therapeutic role of quercetin could improve the lung tissue restoring. Therefore, our experimental results suggested that quercetin might potentially be a protective agent for cyclophosphamide induced lung toxicity. In addition, the promising activity of quercetin as antitumor against lung cancer cells. Prevention of lung cancer with natural extracts as quercetin is considered as a good therapeutic strategy for treatment of human lung cancer and hopeful strategy for replacing chemotherapy with natural sources. In addition quercetin showed potential effect in reducing oxidative stress.

## Supplementary Material

Supplementary data.

## Figures and Tables

**Table 1:** Percentage of cell viability of A549 and H69 cancer cells after treating them with various concentrations of quercetin at different times

Concentration of quercetin (μg/ml)	Percentage (%) of cell viability ± SE						
	A549			H69			W138 (Normal cells)
	24h	48h	72h	24h	48h	72h	48h
**100**	25.12 ± 0.16	21.47± 0.16	20.75± 0.14	30.19± 0.19	26.42± 0.12	22.49± 0.11	57.46± 0.29
**50**	32.82± 0.18	30.52± 0.15	30.14± 0.14	34.28± 0.16	31.64± 0.17	28.13± 0.13	64.19± 0.38
**25**	41.77± 0.23	38.56± 0.18	35.62± 0.17	44.62± 0.19	40.75± 0.17	37.25± 0.15	72.54± 0.45
**12.5**	45.63± 0.24	41.25± 0.22	40.19± 0.20	50.47± 0.24	47.22± 0.22	47.06± 0.21	85.17± 0.49
**6.25**	51.23± 0.26	50.33± 0.25	48.95± 0.23	58.37± 0.31	55.88± 0.28	52.81± 0.27	92.27± 0.57
**0**	100	100	100	100	100	100	100

Each experiment was repeated three times for each concentration.

**Table 2:** Effect of quercetin on apoptosis markers in A549 cancer cells

	Bax conc. pg/ml	Bcl2 Conc. pg/ml
Quercetin	375.22	1.947
Cont. (A549)	51.48	5.058

Each experiment was repeated twice.

**Table 3:** Cell cycle analysis of A549 cells

	%G0-G1	%S	%G2-M	%Pre G1
Quercetin/A549	32.14	27.71	40.15	27.31
cont. A549	50.47	29.66	19.87	2.19

Each experiment was repeated twice.

**Table 4:** Apoptosis analysis of A549/ quercetin cells

	Apoptosis			
	Total	Early	Late	Necrosis
quercetin/A549	27.31	6.37	18.7	2.24
cont. A549	2.19	0.66	0.21	1.32

Each experiment was repeated twice.

**Table 5:** Oxidative parameters evaluation

	GSHP (ng/ml)	SOD (U/L)	MDA (nmol/ml)
Control	0.43± 0.05	0.29± 0.015	0.19± 0.02
Cyclo.	0.15± 0.01	0.13± 0.005	0.41± 0.021
Cyclo. + qur.	0.38±0.016	0.25±0.014	0.20±0.015

Each experiment was repeated three times.
