# Supplementary material for: Antiproliferative, Apoptotic Effects and Suppression of Oxidative Stress of Quercetin against Induced Toxicity in Lung Cancer Cells of Rats: In vitro and In vivo Study
Source: J Cancer. 2021 Jun 26;12(17):5249–59. doi: 10.7150/jca.52088 (PMC8317526; doi:10.7150/jca.52088)
Supplement: Supplementary file 1 — Supplementary data. [file jcav12p5249s1.pdf]

In order to avoid security-related warning messages when switching to secured connection, you may want either to:

- confirm the exception on the next page, or
- import our CA key in your web browser

Click here to proceed.

Click2Drug
SwissDock
SwissParam
SwissSidechain
SwissBioisostere
SwissTargetPrediction
SwissADME
SwissSimilarity

SwissDock

Home | Target Database | Submit Docking | Command Line Access | Help Forum | Contact

Predicted binding modes for your request Quercetinsoddock

This page remains accessible one week after the docking completion. - review parameters

The SwissDock forum can help you understand the docking outcome.

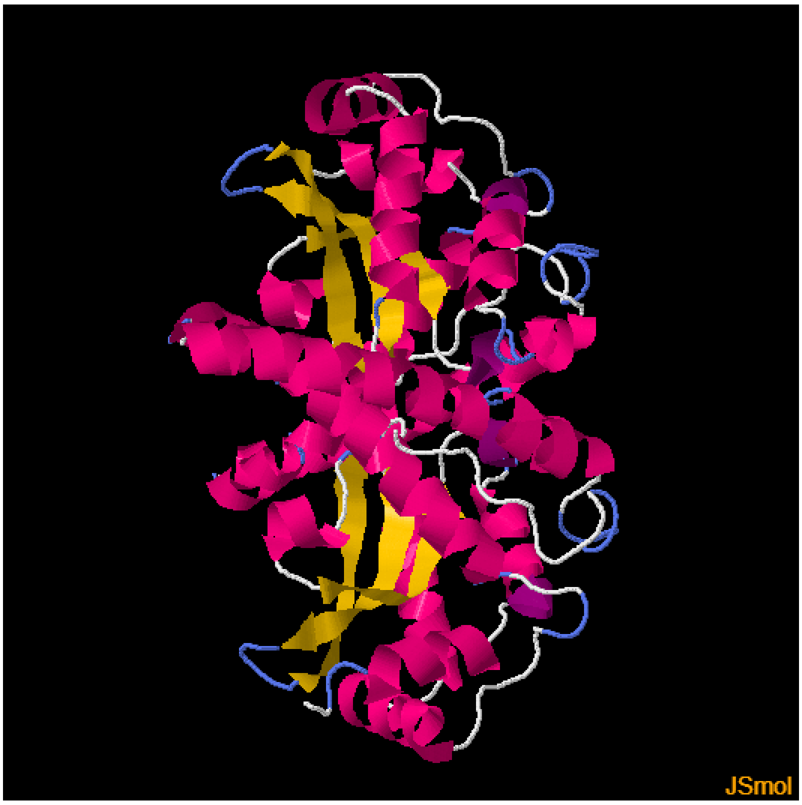

Show	Cluster	Element	FullFitness (kcal/mol)	Estimated ΔG (kcal/mol)
○	0	0	-1880.43	-8.20
○	0	1	-1879.08	-7.97
◉	0	2	-1871.48	-7.31
○	0	3	-1870.86	-7.33
○	0	4	-1870.81	-7.38
○	0	5	-1868.86	-7.37
○	0	6	-1865.51	-7.36
○	1	0	-1878.87	-6.56
○	1	1	-1878.65	-6.54
○	1	2	-1878.25	-6.53
○	1	3	-1878.21	-6.53
○	1	4	-1876.13	-6.30
○	1	5	-1874.69	-6.44
○	1	6	-1874.61	-6.41
○	1	7	-1874.22	-6.56
○	2	0	-1878.77	-7.27
○	2	1	-1877.53	-7.17
○	2	2	-1877.47	-7.17
○	2	3	-1875.99	-6.87
○	2	4	-1875.51	-6.59
○	2	5	-1874.55	-6.64
○	2	6	-1874.54	-6.65

Download CSV file

If predicted binding modes are not displayed, you might suffer from a Jsmol bug we are investigating. In the meantime, please use UCSF Chimera (see below).

Binding modes are scored using their FullFitness and clustered. Clusters are then ranked according to the average FullFitness of their elements (see Grosdidier et. al., Proteins. 2007 Jun 1;67(4):1010-25.

For further inspection, you can either download predictions files, or open UCSF Chimera from your browser:

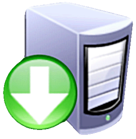

Download your predictions file

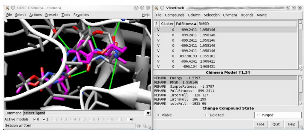

Launch UCSF Chimera to visualize predicted binding modes
You can get Chimera here. Your browser must be properly configured.

Help us improve SwissDock by telling us

Help us improve SwissDock by telling us more about you

This survey is not mandatory. If you choose to answer it, please do it only once.

* Required

What is your email address? *

Your answer

What is your background? *

- ◯ Biology
- ◯ Chemistry
- ◯ Physics
- ◯ MD
- ◯ PharmD
- ◯ Other:

What best describes your current position? *

- ◯ Student
- ◯ PhD
- ◯ Post-Doc
- ◯ Permanent position
- ◯ Professor
- ◯ Other:

What are you using SwissDock for? *

- ◯ Creating pictures
- ◯ Testing hypotheses
- ◯ Finding active compounds
- ◯ Other:

Which molecular viewer are your familiar with? *

- ☐ UCSF Chimera
- ☐ PyMol

- [ ] VMD
- [ ] Other:

Are you familiar with scripting (bash, perl, etc...)? *

- ( ) No

Like 426 people like this. Sign Up to see

This server is free for academic use. A CHARMm license is required for users from private companies.
Please contact us if you need your own private SwissDock service.
